# Paget disease of bone in an elderly patient with chronic renal disease and weight loss: A case report

**DOI:** 10.1097/MD.0000000000017458

**Published:** 2019-10-18

**Authors:** Po-Kai Chan, Sin-Yi Lyu, Chun-Chi Lu

**Affiliations:** a Department of General Medicine, Tri-Service General Hospital, National Defense Medical Center, Taipei; b Department of Radiology, Taipei Medical University-Shuang Ho Hospital, Ministry of Health and Welfare, New Taipei City; c Department of Internal Medicine, Division of Rheumatology, Immunology and Allergy, Tri-Service General Hospital, National Defense Medical Center, Taipei, Taiwan; d Department of Pathology, University of Washington, Seattle, WA.

**Keywords:** metabolic bone disease, metastatic bone disease, mixed osteolytic and blastic lesion, Paget disease of bone, radiography interpretation

## Abstract

**Rationale:**

Asymptomatic Paget disease of bone (PDB) is mostly diagnosed by accidental finding of osteolytic lesion on the plain film. However, in elderly patient with chronic renal insufficiency and weight loss, it is crucial to differentiate PDB from metabolic and metastatic bone diseases for further treatment and better outcome.

**Patient concerns:**

An 80-year-old man with chronic kidney disease presented to our emergency department due to fever with chillness for a day, while the abdominal fullness, anorexia, and weight loss had been noted for 3 months. Mixed osteoblastic and lytic changes in the pelvic bone were accidentally found on the abdominal plain film.

**Diagnosis:**

The patient was diagnosed as asymptomatic PDB and urinary tract infection of *Pseudomonas aeruginosa*.

**Interventions and outcome:**

The patient received 7 days intravenous and followed by 7 days oral antibiotic treatment, which lead to clinical improvement of his urinary tract infection. No pharmacological treatment was initiated for the asymptomatic and localized PDB. The patient was discharged under stable condition afterward.

**Lessons:**

In patients with mixed osteolytic and blastic lesions, the differential diagnoses include metabolic and metastatic bone disease. Thorough understanding of the morphology of the bone lesions in high risk patient, not only helps to make differential diagnosis, but it also leads to precise treatment and better outcome.

## 1 Introduction

Paget disease of bone (PDB) is mostly asymptomatic and is detected on imaging studies that were performed for some other reason.^[1]^ Compared with the western country, the prevalence of the Paget disease is low in Asia.^[1]^ When it comes to those with chronic renal disease, the prevalence was unknown and only 6 cases in renal replacement therapy were reported having the PDB.^[2]^ We presented a challenging and educational case of PDB in an elderly patient with chronic renal disease and weight loss. The patient has provided informed consent for publication of the case.

## 2 Case presentation

**Figure 1:**
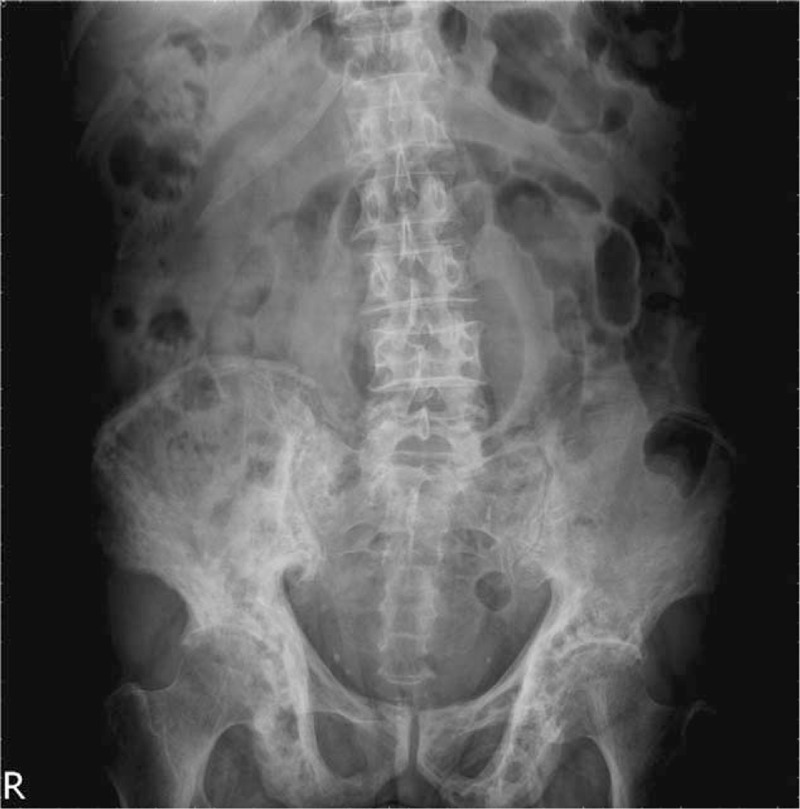
The abdomen plain film with mixed osteoblastic and osteolytic changes in the pelvic bone.

An 80-year-old man had already been diagnosed stage V chronic kidney disease for 2 years but did not receive renal replacement therapy. Otherwise, he had no history of foreign travel and familial problems. This time, he visited our emergency room due to high fever and chills for a day. Abdominal fullness, anorexia, and weight loss had also developed for 3 months before this visit. The physical examinations revealed a tachycardia of 114 beats per minute, fever of 38.4°C, and diffuse abdominal tenderness. The blood sampling showed leukocytosis, azotemia, and increased serum procalcitonin level (Table 1). The urine analysis revealed the pyuria, suggesting the complicated urinary tract infection. The abdominal plain film showed mixed osteoblastic and osteolytic changes: cortical thickening, sclerosis with coarsened trabeculae, and flame-shaped lucent lesion in the pelvic bone with right site predominantly (Fig. 1).

**Table 1:**
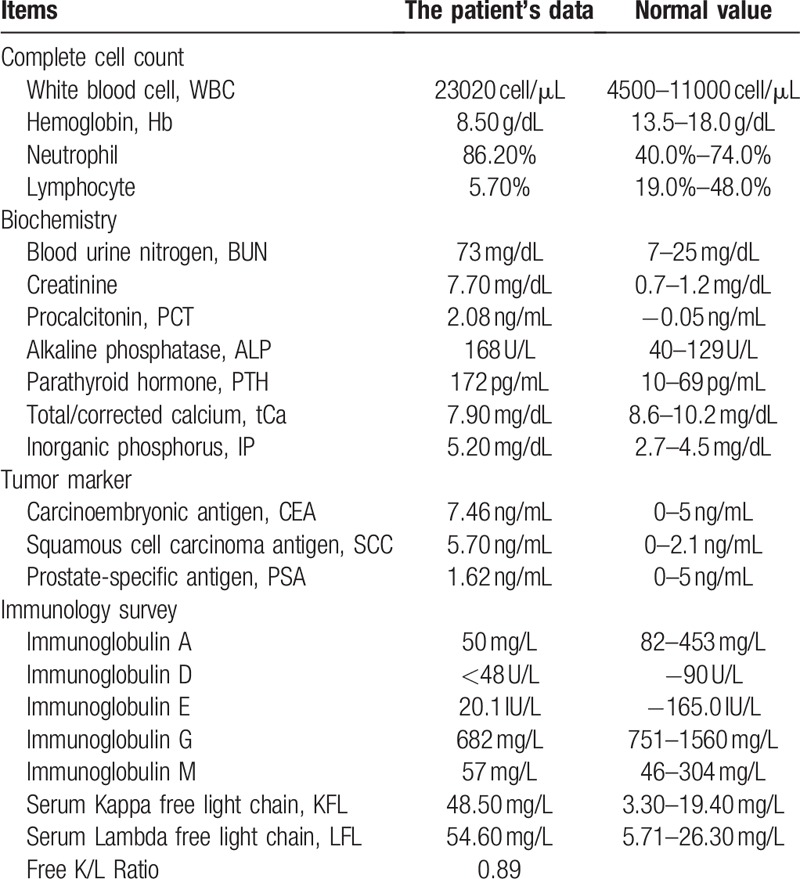
The laboratory data.

Considering the image results and other clinical symptoms such as anorexia and weight loss, the differential diagnosed of the bone lesion is crucial for the patient. The blood chemistry results on the second day, showed only mild elevation in alkaline phosphatase (ALP) and inorganic phosphorus, while highly elevated parathyroid hormone but decreased corrected calcium was noted, favor secondary hyperparathyroidism due to chronic renal function insufficiency (Table 1). On the third day after admission, we check the serum tumor markers of leading causes for bone metastasis in the male patients, such as carcinoembryonic antigen and squamous cell carcinoma antigen for lung cancer and prostate-specific antigen for prostate cancer, and all of 3 markers showed normal or mild elevation (Table 1). Monoclonal globulin elevation was not identified in serum screening as well (Table 1).

**Figure 2:**
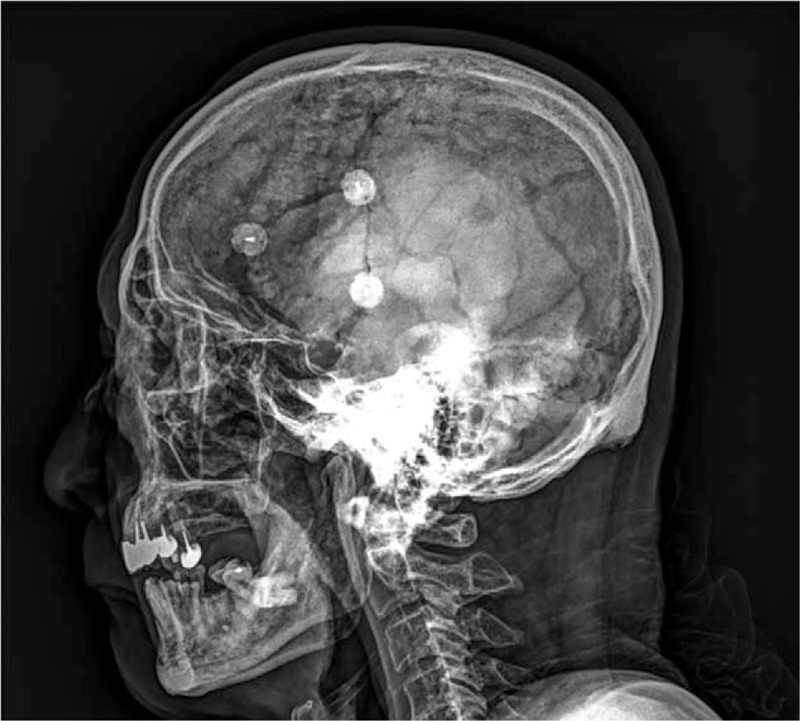
The skull X-ray plain film demonstrated no osteolytic lesions.

**Figure 3:**
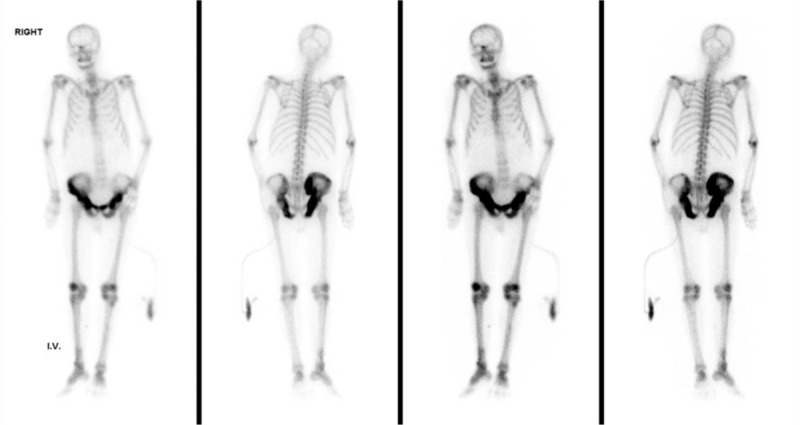
The total body bone scintigraphy disclosed no lesions throughout the entire body except for the pelvic bones.

Otherwise the abdominal plain film, the skull X-ray plain film was performed on the second day after admission and demonstrated no osteolytic lesions (Fig. 2). We arranged gallium-67 tumor scan and Tc-99m methylene diphosphonate bone scan on the third day and disclosed no lesions throughout the entire body except for the pelvic bones (Fig. 3).

The patient was diagnosed as asymptomatic PDB based on the pelvic X-ray and elevated ALP level without extended high-risk bone involvement on bone scan, and complicated urinary tract infection of *Pseudomonas aeruginosa*. He received intravenous hydration and piperacillin 4 g and tazobactam 0.5 g injection every 12 hours, which led to the clinical improvement of his urinary tract infection after 7 days treatment. We shifted to ciprofloxacin 500 mg orally once per day for another week and discharged the patient. A week later, the patient visited our outpatient department with complete recovery from infection and had no symptom of PDB such as bone pain, arthritis, deformity, or fracture.

## 3 Discussion

In patients with osteolytic and osteoblastic lesions, the differential diagnoses include metabolic bone diseases and metastatic bone disease. Metabolic bone diseases such as hyperparathyroidism, osteomalacia, and PDB share similar laboratory findings but have varied clinical manifestations and different features on imaging. Those with hyperparathyroidism may develop the radiolucent lesions in the phalanges, a salt-and-pepper appearance in the skull and subchondral resorption in the iliac bones of the sacroiliac joint in the X-ray.^[3]^ Painful fracture lines, or looser's zones, are often noted in the long bones of patients with osteomalacia.^[3]^ The typical imaging features of PDB include the wedge-shaped radiolucent areas resembled a “blade of grass” or “ flame” caused by increasing bone absorption in the early stage. Simultaneously, the new sclerotic bone develops in response to osteolytic activity and replaces the normal lamellar bone with woven bone. Eventually, mixed lytic and sclerotic areas lead to the characteristic appearances of thickened trabecula, bone expansion, cortical thickening, and deformity.^[1]^

In addition to metabolic bone disease, for which radiography discloses osteolytic lesion only or mixed type with osteoblastic lesions, the differential diagnoses should include metastatic bone diseases of solid tumors or blood cancer. Solid tumor such as breast, lung, and prostate cancer often cause unbalanced bone remodeling and lead to the different degree of osteolytic or osteoblastic activities. On bone scan image, metastatic disease may present with asymmetrical and spotty uptake, while, in contrast, our case presented typical homogeneous tracer uptake of the PBD that is intense, symmetrical, well-demarcated, and anatomic configuration preserved of the involved bone.^[4]^ The multiple myeloma is characterized by elevated osteolytic activity and suppression of osteoblast, while limited cases had reported mixed lytic and blastic lesions.^[5,6]^

It is challenging to differentiate PDB from metabolic bone diseases and metastatic bone disease based on clinical manifestations and laboratory samplings, especially in an elderly patient with chronic renal insufficiency and weight loss. Elderly patients with renal impairment usually suffer from osteoporosis and chronic kidney disease-mineral and bone disorder (CKD-MBD).^[7]^ The pathogenesis of CKD-MBD involves numbers of physiology loop between the kidney, parathyroid glands, and bones. Even in subclinical stage, secondary hyperparathyroidism-related CKD-MBD may result in high or low bone turnover rate and thus mask the diagnosis of PDB.^[7]^ Bone biopsy is the golden standard to differentiate the diagnosis between CKD-MBD and PDB, but for its invasive risk, the 2017 The Kidney Disease: Improving Global Outcomes (KDIGO) guideline no longer suggests routine performance.^[7]^ In our case, we identified the typical imaging features of PDB based on the pelvic plain film with no skeletal involvement other than iliac bone detected by scintigraphy. Although the secondary hyperparathyroidism was compatible with the laboratory findings and the history of chronic renal impairment, the images of the skull and pelvic bones had no corresponding features.

In addition, advanced age with weight loss and anorexia remind us the possibility of malignancy; however, the metastatic bone disease from the solid tumors was not likely for our patient due to negative finding in serum tumor markers and well-demarcated lesion confined to the pelvic bone on bone scan. The renal function impairment, anemia, and osteolytic lesions are typical manifestations of multiple myeloma; nevertheless, the sclerotic bone lesion, no hypercalcemia, and no predominant monoclonal serum plasma cell or protein made the diagnosis less feasible for this patient.

In asymptomatic patients, regular serum total ALP concentration surveillance and the total body bone scintigraphy provide useful information for the disease activity and the existence of high-risk extensive bone involvement respectively.^[8]^ If active disease involves the sites at the high risk of late complication, such as long bone at risk of further bowing deformity, skull with potential loss of hearing, and vertebrae with the risk of future neurological complication, should receive pharmacological treatment.^[7]^ Oral or intravenous bisphosphonates is the main treatment choice for PBD, but nonetheless there was limited literature discussing about the treatment for those with both PBD and CKD or malignancy. The 2017 KDIGO guideline suggested aggressive control of serum parathyroid hormone, calcium, phosphorus, and vitamin D level for the CKD-MBD patient.^[9]^ If treatment of bone with bisphosphonates is required for renal osteodystrophy, their specific side effects and nephrotoxicity must also be considered.^[7]^ On the other hand, the PDB with coexisting or subsequently solid tumors or metastases were seen in previous case reports, but treatment for PDB in those cases were scarcely discussed.^[4,9]^ Nevertheless, since the solid tumors are at risk of bone metastases and further bone complication, bisphosphonates and denosumab have been shown to prevent bone loss and reduce the bone pain in several researches and guidelines.^[10]^

In our case, due to the early stage of the asymptomatic PDB without the existence of high-risk extensive bone involvement, we did not initiate pharmacological treatment after discussion with the patient and his family. However, due to the advanced age and high risk of further complication, the patient was encouraged to follow up closely and initiate treatment for CKD-MBD or PBD if associated symptoms or high-risk bone involvement developed.

## 4 Conclusion

In an elderly patient with chronic renal insufficiency and weight loss, the PDB, other metabolic bone diseases, and metastatic bone diseases shared similar clinical manifestations and laboratory samplings. Thorough understanding of the changes in bone morphology on imaging examination associated with Paget disease not only helps to ensure an accurate diagnosis but also prevents further high-risk extensive bone involvement and lead to better outcome.

## Author contributions

**Conceptualization:** Sin-Yi Lyu.

**Investigation:** Sin-Yi Lyu.

**Supervision:** Chun-Chi Lu.

**Validation:** Sin-Yi Lyu.

**Visualization:** Sin-Yi Lyu.

**Writing – original draft:** Po-Kai Chan.

**Writing – review & editing:** Chun-Chi Lu.
